# Psychosocial aspects of identity-release gamete donation – perspectives of donors, recipients, and offspring

**DOI:** 10.1080/03009734.2019.1696431

**Published:** 2019-12-05

**Authors:** Agneta Skoog Svanberg, Gunilla Sydsjö, Claudia Lampic

**Affiliations:** a Department of Women’s and Children’s Health, Uppsala University, Uppsala, Sweden;; b Department of Obstetrics and Gynaecology and Department of Clinical and Experimental Medicine, Linköping University, Linköping, Sweden;; c Department of Women’s and Children’s Health, Karolinska Institutet, Stockholm, Sweden

**Keywords:** Disclosure, donor offspring, gamete donation, open-identity donors

## Abstract

Donor conception creates families with varying genetic linkage between family members. This may have short-term as well as lifelong psychosocial consequences for all involved. Gamete donors have traditionally been anonymous to recipients and offspring, but there is a growing trend towards identity-release donor programmes that give offspring the right to obtain the donor’s identity. This review aims to provide an overview of the perspectives of donors and recipients and offspring involved in identity-release donation. The results show that both oocyte and sperm donors have primarily altruistic motives, and recipients, in particular lesbian and single women, are generally open about the donation to their offspring. The few existing studies on offspring perspectives indicate that those who are aware of their donor conception appear to be interested in contact with the donor, and most donors are open to such contact. Investigations of donors and recipients indicate a need for more counselling and support to manage family life with varying genetic linkage within and outside the family unit. This includes preparing for and managing future contact between the donor and his/her family and donor offspring and their family, as well as between donor siblings and their respective families.

## Introduction

The desire to have a child is based both on psychological and social reasons. Assisted reproduction treatments have made it possible for many individuals/couples to have a family even when sperm and oocytes are reduced or lacking. Large numbers of children are born following donor conception, and there is an increasing demand for donor conception worldwide. Depending on legislation and regulations, treatment with donor sperm, oocytes, and embryos is available to various groups of recipients, including heterosexual couples, lesbian couples, and single women. Donor conception has traditionally been performed with anonymous donors, while it has been less common to use a donor who is ‘known’ to the recipient(s), most often a female relative who donated oocytes. There is a global trend towards programmes using donors that are identifiable to the resulting offspring at maturity, commonly labelled ‘identity-release’ or ‘open-identity’ donors. The use of identity-release donors implies that the donor is anonymous to the recipients, although they may receive some non-identifying information about the donor. Upon request from a donor-conceived child that has reached mature age, the donor’s identity is released to the child. Legislation on identity-release gamete donation was first introduced in 1985 in Sweden (1) and later in other jurisdictions (e.g. Austria, Switzerland, New Zealand, and the UK).

Donor conception creates families where the genetic linkage between family members is varying and where there are genetic links to individuals outside the family unit. The presence and/or absence of genetic linkage may have psychosocial consequences for all involved parties, i.e. for the donor and his/her family, as well as for the recipient(s), the donor-conceived child, and their larger family. There is a relatively large body of research on the psychosocial aspects of anonymous donor conception, and to a lesser extent on ‘known’ donation. Systematic reviews in the field show that both oocyte and sperm donors’ psychosocial wellbeing was good throughout all donor groups (anonymous, known, egg-sharers, and open-identity) (2,3), and families created through the use of donor gametes appeared to be well-adjusted (4). During recent years, the attitude towards disclosure in gamete donation has shifted from secrecy to openness, and disclosure is now strongly encouraged by the Ethics Committee of the American Society of Reproductive Medicine (5). A recent systematic review focussing on donor-conceived offspring showed that genetic ties are perceived as important, especially during adolescence and adulthood, and that many were interested in receiving more information about the donor and for potential contact (6). In contrast to the relatively large body of research on anonymous and known gamete donation, research on the psychosocial aspects of conception with gametes from identity-release donors is more limited, mostly due to the fact that this type of donor conception has been less common.

The purpose of the present review is to summarize the available research on psychosocial aspects of identity-release gamete donation. Furthermore, we want to present an overview of the specific perspectives of donors, recipients, and offspring, including motivations for participating in this type of donor conception, as well as perceptions of disclosure issues and potential contact between donor and offspring/family. PubMed and PsycInfo databases were used in order to search for relevant empirical studies. Due to the diverse use of terminology for this type of donation (e.g. open-identity, identity-release, open donation) it is possible that some studies including relevant groups were missed. This overview covers only studies that specifically noted the inclusion of donors, recipients, or offspring involved in identity-release donation, but does not include studies on surrogacy, although such arrangements frequently include donor gametes. In several studies, a small subset of participants concerned identity-release donation. Such studies were included provided that they presented results from these groups separately, and were otherwise excluded [e.g. (7,8)]. Not surprisingly the largest number of identified studies was performed in Sweden, where identity-release gamete donation has been mandatory since 1985, and the remaining studies were conducted in other European countries and in the USA.

## Perspectives of identity-release donors

### Motives and characteristics

By definition, the term donation implies altruism, and both male and female donors who agree that their identity may be released to offspring at maturity have been found to donate primarily or solely based on altruistic reasons, i.e. they want to help involuntarily childless people (9–13). However, also other motives such as receiving confirmation of one’s own fertility potential, spreading one’s good genes, and donating as a way to have a child/children in the future have been reported (11,14,15), with sperm donors more often reporting spreading their genes as a salient motive compared to oocyte donors (11). Although the dominant motive for donating was altruistic, two small survey studies found that a subgroup of identity-release sperm donors also reported financial motives (9,10). A Danish interview study with sperm donors, five of whom had opted for identity-release status, found that financial compensation was a factor for the decision to allow more information about them to be made available to recipients, but the child’s wellbeing was also considered when providing extended information about oneself (15). Also, in a large Finnish study of oocyte donors, one in four reported that the financial compensation had at least some influence on their decision to donate (13). In contrast, in a recent interview study including 24 oocyte and sperm donors in the UK, all rejected the idea that they had been financially motivated, and it was particularly important for sperm donors to frame their donation as a purely altruistic ‘gift’, as a financial motive was perceived to be incompatible with a beneficial potential relationship with offspring from their donation (16). Oocyte donors, on the other hand, were more comfortable to incorporate the fact that they received financial compensation in the narrative of their donation as a ‘gift’ to recipients longing for a child.

The personalities and characters of gamete donors are of interest for the recipients but also for the resulting offspring. In a Swedish national study, the Temperament and Character Inventory (17) was used to assess both oocyte and sperm donors. The sperm donors were found to all be in the normal range of character, which means that the donors perceived themselves as autonomous individuals with capacity to take responsibility and with the ability to behave in a goal-oriented manner (18). The sperm donors described themselves as persons well integrated in society and having a capacity for relatively high identification with and acceptance of other people. Concerning the personality and character of the oocyte donors, it was evident that they described themselves as less worried, shy, and fatigued, and as more persistent compared to a comparison group of women in fertile age (19). These findings are reassuring for all involved, both in the donation and treatment process, and for the future families, and suggest well-functioning screening procedures in a non-commercial donor programme.

### Openness about donating

Little is known about donors’ view of informing others, such as partners and biological children, about their donation. In two Swedish survey studies, each including 30 sperm donors, almost all had shared information with their partner about their intention to donate (10) or of being a donor (20). The involvement and support from the partner seemed to be important factors for the decision to donate sperm, particularly among younger men (9). Many donors in the Ekerhovd et al. (10) study planned to inform their own children of the donation, particularly if the donation did result in a child. Similarly, in a well-designed Finnish study, almost all oocyte donors who were mothers either had or planned to share this information with their children (21).

### Thoughts about offspring and potential contact

For identity-release gamete donors, the number of children conceived with their gametes is of particular interest, since the offspring will be able to obtain the donor’s identity and may attempt to contact the donor. However, only one study was found investigating donors’ views regarding the number of children a donor may conceive (22). About half of 235 oocyte and sperm donors 5–8 years after their donation regarded 1–10 children to be an acceptable number of offspring from one donor, with oocyte donors more often supporting an upper limit than sperm donors. Following identity-release donation, a majority of both oocyte and sperm donors would like to be informed if their donation results in pregnancy and birth (10,23). In a Swedish follow-up study of gamete donors, sperm donors reported a higher level of emotional involvement with offspring from their donation compared to oocyte donors (23). This included wanting to know how the child fares in life and feeling responsibility for the child if anything happened to his/her parents.

In two Swedish qualitative studies, each including 30 sperm donors, most of them were positive or neutral towards contact with adult offspring from their donation (10,20). This finding was confirmed in a large follow-up study of sperm donors and oocyte donors 5–8 years post-donation (23,24). Ten percent of donors were negative towards being contacted by an offspring, and some comments indicate that this was based on a desire that the child would feel no need for contact and be ‘happy in their real family’ (24). Among oocyte donors in Finland, a majority stated that they were positive or neutral towards future contact with an offspring, but they were more uncertain regarding potential contact between their own children and a donor offspring (21). Furthermore, in a qualitative study of 11 oocyte donors from the UK, women were happy to be contacted by offspring, but some expressed concerns regarding potential negative impact of such contact on the offspring’s parents and on the donor’s own family (25). So far, only one study has reported on identity-release donors’ position when adult offspring from their donation seek information about them (26). In that study, from one sperm bank in the USA, clinic staff contacted sperm donors when information about them was being requested, and 39 out of 43 men responded that they were open for contact with their offspring.

### Satisfaction with the donation and need for counselling

The experiences, satisfaction, and consequences of being an identity-release gamete donor, i.e. with respect to the medical care and treatment, have been investigated in a few structured follow-up studies. In a Finnish follow-up study of 428 former oocyte donors, 67 of which were identity-release, almost all were satisfied with their donation (13). Similarly, in a Swedish study of 300 oocyte and sperm donors, most of them were satisfied with the donation (11). Those who expressed ambivalence before the donation (but after being accepted in the programme) reported lower satisfaction 2 months after their donation (27).

In a qualitative study from the Netherlands, male donors expressed a need for counselling in order to discuss the emotional consequences of their donation, disclosure to their own children, family, and friends, and potential future contact with an offspring (28). In a Swedish follow-up study of 210 oocyte and sperm donors several years following donating, one in four donors reported a need for counselling about how to manage potential future contact with offspring from their donation (24). More than half of these donors wanted to be notified when an offspring requested information about them in order to prepare for potential contact, while one-third were negative to receiving such information, partly to avoid potential disappointment if no contact attempts would follow. In the above-mentioned Finnish study, the oocyte donors indicated a high level of satisfaction with the support offered during the process and a relatively low need for additional support (13).

## Perspectives of recipients

### Motivation for choosing identity-release donation

In general, reasons for choosing treatment with donor gametes include having a biological tie to the child, desiring to experience a pregnancy and to have a child who has a genetic link to at least one parent. The most common reasons for choosing identity-release sperm donation stated by heterosexual-couple, lesbian-couple, and single-woman parents in a US study were that this gave the child the option of getting more information about the donor, including his identity, and the option to be able to meet him (29). In a US study of 129 lesbian mothers, most of them were satisfied with their choice of an anonymous, known, or identifiable donor (30). Those who had selected an open-identity donor were most satisfied with their choice, both because they avoided potential custody conflicts and/or involvement from a third person, and because offspring would have access to information about the donor.

### Disclosure

Identity-release donation gives the offspring an option to obtain identifying information about the donor. However, offspring can only make use of this option if he/she has been made aware of the donor conception, most often by his/her parents. In a Swedish study of 148 heterosexual couples with children conceived through donor insemination in the years directly after the law was introduced, only 11% had informed the child about the donor conception, 46% planned to disclose later, and one-third were unsure or planned not to disclose (31). Those who had disclosed did not regret their decision to disclose and thought that being open about the donor conception had been beneficial to the child. In a follow-up interview study including 19 couples from the above study, participants said that healthcare staff had influenced their thinking, and a majority of those who had been encouraged to tell their children about the donation had done so (32). Another interview study of 31 heterosexual-couple parents with 1- to 7-year-old children conceived with donor sperm during 1997–2003 found that 75% already had or planned to talk with the child about the donation (33). In a later Swedish study of 111 heterosexual couples with 1- to 4-year-old children following oocyte or sperm donation (34), 78% planned to disclose to the child about the donation, and 16% had already started the disclosure process. A subset of 30 heterosexual sperm recipients from that study also participated in an interview study when the child had reached 7 years of age (35). The authors concluded that sharing information about donor conception with offspring was a complex process that involves different levels, and in which parents’ beliefs and the child’s responses serve as driving or impeding forces.

A UK study investigated disclosure to offspring among 31 heterosexual solo mothers and 47 heterosexual-partnered mothers with donor-conceived children aged 4–8 (36). About half of the solo mothers and one-third of the partnered mothers had already told the child about their conception with donor sperm. Among those who had not yet disclosed, partnered mothers were significantly more reluctant or negative to disclose than solo mothers. In line with this finding, a US study showed that all single women and lesbian couples with adolescent offspring had disclosed their use of donor insemination to their children, while this was the case for 70% of heterosexual couples (29). Disclosure did not seem to have any negative impact on the families, regardless of the parents’ sexual orientation or relationship status.

Several studies have found that large groups of recipients had told other persons about their use of gamete conception (32–34,37–39), with no differences in disclosure behaviour with regard to sex or type of donation (oocyte/sperm) (34). One stated reason to refrain from sharing this information with people outside a close circle of friends and family was that the child should learn about the donation before other people did (34). Among parents who plan not to share information about the donor conception with their child, disclosure to others increases the risk of accidental disclosure.

### Managing family life

When a couple conceives with donor oocytes or sperm, this creates a family where the child has a genetic link to only one of the parents. The presence and/or absence of genetic linkage, as well as the existence of an identifiable donor, may have psychosocial consequences for the couple and the family. Two Swedish interview studies of heterosexual couples following sperm donation concerned family life. Leeb-Lundberg et al. (33) reported that some parents had worried that the lack of a genetic link to the father would create an unequal relationship with the child, but these concerns had disappeared over time. Isaksson et al. (40) found that resemblance between child and parent was an important theme, and parents were reported to navigate between the importance of genetic connectedness and of ‘doing parenthood’ through social interactions. Non-resemblance between parent and child was described to bring the donor to the front and the donor constituted an ‘absent presence’, as also described in a qualitative study of single mothers in the UK (41).

In the longitudinal ‘Swedish Study of Gamete Donation’, recipient couples of donor oocytes and sperm were followed 2–5 years after treatment. Heterosexual couples that had been treated with sperm donation expressed satisfaction with their relationship (42), and couples using oocyte donation treatment had a balanced and solid view of their relationship (43), where having children or not after treatment had no effect on the nature of the relationship. Lesbian couples following sperm donation reported stable relationships and a high satisfaction with their relationship, also after unsuccessful treatment (44). They also reported less parenting stress compared to heterosexual-couple parents following IVF with their own gametes and parents following a spontaneous pregnancy (45).

While a previous study in the USA indicates that, following donor insemination, heterosexual couples, lesbian couples, and single women were positive about their decision and were quite open about the donor conception (29), lesbian-couple families may face specific challenges related to their non-traditional family formation. Results from interviews with 20 female-partnered mothers of young children indicate that participants had lacked psychological support in the process of planning and becoming a parent (46). They expressed a desire to be treated as equally valid mothers and as a proper family by professionals at child healthcare services (47). Also, the results from a web survey with 145 Belgian and Swedish participants, 36 of whom had used identifiable donors, showed that donor-conceived families were challenged by cultural norms and values and responses from friends, healthcare professionals, and teachers (39).

When children conceived with gametes from identity-released donors grow up, parents need to deal with the question of disclosure to the child. In a study of 111 heterosexual couples following oocyte and sperm donation, one-third were not in agreement about what to disclose to their child about his/her conception, and these couples reported a lower relationship quality than couples who agreed about disclosure (34). However, incomplete couple agreement about disclosure did not appear to have a negative impact on parental stress (48).

### Contact with donor and donor-siblings

In a Swedish study including 279 heterosexual recipient couples of donor oocytes or donor sperm, about half believed that it was in the best interest of the child to be able to obtain identifying information about the donor, while the remaining were unsure, neutral, or negative (37). While few believed that contact between the child and the donor could be harmful for the offspring or family, about one-third could not form an opinion about this. In a subsequent qualitative study of 30 heterosexual parents with a 7-year-old child, some parents were curious about the sperm donor and hoped that the child would make contact in the future, while other parents expressed concerns about potential contact between the child and the donor (35). With openness about donor treatment, both parents and offspring may want to get information and contact others who share the same donor. The Sperm Bank of California has established a service that connects families who share the same donor, and it has been used predominantly by families headed by lesbian couples and single women, most of whom had used identity-release donation (49). These groups’ motivations for contact with other families who share the same sperm donor included to create a family for the child, obtain support for their children and/or themselves, and to get information about shared traits and medical problems (49,50). Female-partnered women most often described their own and their children’s relationship with ‘linked’ families as a unique type of relationship, a ‘special bond’, an ‘extended family’, or merely ‘acquaintances’ (51).

## Perspectives of offspring

There is a dearth of research including the perspectives of persons conceived with gametes from donors who originally chose to donate within an identity-release programme, and all concern persons conceived with donor sperm. Scheib and co-workers have presented several studies based on donors, recipients, and their offspring from one sperm bank that has offered identity-release donors since 1983. One interview study included 29 adolescents conceived with sperm from open-identity donors, from households led by lesbian couples, single mothers, and heterosexual couples (52). A majority (76%) reported always knowing about their donor conception and were comfortable with their conception origins. Most also planned to request the donor’s identity and pursue contact in order to learn more about themselves. These results are in line with two longitudinal studies of families headed by lesbian couples in the Netherlands and the USA, where half of adolescents conceived with sperm from an open-identity donor reported a desire to meet their donor (53) and 67% of adolescents planned to contact the donor at the allowed age of 18 (8).

Only one study was found that reported on the final step of an identity-release donor programme, i.e. requests and provision of identifying donor information to adult offspring. In a follow-up study, Scheib et al. (26) reported on the first 10 years of this practice at one sperm bank. During this period, adult offspring from 256 families were eligible to receive such information, and a total of 85 offspring (35%) contacted the clinic for this purpose. Being a female offspring and belonging to a single-parent household increased the probability of requesting donor information, while having heterosexual-couple parents decreased the likelihood of a request. A large majority of offspring contacted the clinic for information within the first three years after turning 18. The most common motivations for requesting the donor’s identity were to gain information about who the donor is as a person, his motives for donating, and medical or health information. Many believed that information about the donor would help them learn something about themselves and help to ‘fill in the missing links’. While a majority (75%) expressed an interest in contacting the donor, most had low or no specific expectations of a potential contact, and very few expressed a desire for a close relationship. Four offspring were informed by the clinic that their donor was not open to contact and were reported to be very disappointed and upset.

## Discussion

The aim of this review was to provide an overview of the perspectives of donors, recipients, and offspring involved in identity-release donation. Identifiable donors of oocytes and sperm predominantly reported altruistic motives, in line with motives reported for anonymous and known donors (2,3). Still, the prospect of a potential future meeting with donor offspring may influence how donors reflect about and frame their motives for donating. Identity-release oocyte and sperm donors were found to be mature and well-adjusted individuals (18,19), which is reassuring and indicates that the screening procedures are well-functioning. The studies that have investigated long-term consequences of donating in an identity-release donor programme indicate that most donors were satisfied with their decision (11,13) and had positive or neutral attitudes towards being contacted by offspring from their donation (21,23,24). However, subgroups of donors expressed a need for support and counselling, both to handle their own situation and to prepare for a potential situation when an offspring seeks contact (or not) (24,28). While a recent review concluded that families following gamete donation in general are well-functioning (6), conception with oocytes or sperm from a donor who will be identifiable to the child at maturity may have specific psychosocial consequences. The present results indicate that recipients of gametes from identity-release donors had stable relationships (42–45) and were increasingly open about having used donor conception (33,34), with female-partnered and single women being most positive towards disclosure to the offspring (29,36). Parents were generally satisfied with their choice of an identity-release donor as this gives their child the option to obtain more information about their genetic origin (29,30). Some parents had own interest in contact with the donor and/or with families who had used the same donor (49,50).

Concerning the perspectives of offspring conceived with gametes from identity-release donors, the present review highlighted the very limited knowledge base for this specific group. Only four studies were found (8,26,52,53), all concerning adolescent and young adult offspring conceived with donor sperm. The results indicate that about half of the offspring who are aware of their donor conception, and have the possibility to obtain the identity of their donor, plan to do so, and many also intend to contact the donor. Their motivations for learning the donor’s identity and meeting the donor are in line with those reported by offspring conceived with gametes from anonymous donors (6). The study by Scheib et al. (26) is the first to report on the percentage of offspring eligible to receive identifying donor information who actually made such a request, which was about one-third of the total sample. Also, it was recently reported that only 5% of eligible adult offspring from heterosexual-couple families in Sweden had requested donor information (54). At the moment, there is no information available about the large groups that have not (yet) requested information about their donor. Are they aware of their donor conception and the possibility to obtain the donor’s identity? What is their level of interest in this information? Are there any practical issues, concerns, or considerations that impact their decision not to seek donor information? In view of the relatively low disclosure rates reported by heterosexual couples who conceived with donor gametes in the 1980s and 1990s (i.e. whose offspring have reached adulthood by now) (55), is it reasonable to assume that at least some of those offspring are unaware of their origin with donor gametes.

The studies covered in the present overview were performed in Europe and the USA and included quantitative and qualitative studies with cross-sectional, retrospective, and longitudinal designs. Studies of individuals involved in gamete donation are frequently based on single clinics and suffer from relatively low response rates, which limits the generality of the findings. Research on donor-conceived offspring is hampered by specific difficulties identifying this population, as it is ethically only possible to approach individuals who are aware of their donor conception. Recruitment of participants through self-selection, e.g. membership in networks for donor conception, is feasible but introduces a selection bias. Thus, there is a lack of knowledge about the perspectives of donors, recipients, and offspring who are not interested in seeking information about genetically related persons. Also, the number of long-term follow-up studies in this field is limited, and attrition is a matter of concern, as in all longitudinal designs.

Changes in legislation, DNA-based voluntary contact registers, and direct-to-consumer genetic tests constitute new challenges and may have great influence for the future for assisted reproduction with donor gametes. The possibility of identification of genetically related individuals may have an impact not only on donors, recipients, and offspring, but also on their extended families (56). The need to ensure that all this information is handled with the best safety and privacy rights has been stressed (57). However, reflections on the future of genetic testing and/or screening must be distinguished from the long-standing debate about disclosure of donor conception to children (58).

In conclusion, donor conception creates families with varying genetic linkage between family members and where there are genetic links to individuals outside the family unit. In the case of identity-release donation, the offspring has the opportunity to obtain identifying information about his/her donor. Existing research about the perspectives of donors and recipients involved in identity-release donation indicates that both oocyte and sperm donors primarily have altruistic motivations and that recipients are increasingly open about having used donor conception. Furthermore, several studies indicate that the offspring are interested in contact with the donor, and most donors are open to such contacts. Keeping in mind the lifelong consequences of identity-release donor conception, recipient families and donors could benefit from support and counselling to increase their confidence in managing family life. In view of the present findings, as well as the rapid development and increasing use of resources to identify genetic relatives, more high-quality research is warranted on the long-term psychosocial consequences of gamete donation.
